# A Systematic Review about the Efficacy and Safety of* Tripterygium wilfordii* Hook.f. Preparations Used for the Management of Rheumatoid Arthritis

**DOI:** 10.1155/2018/1567463

**Published:** 2018-02-08

**Authors:** Jing Wang, Na Chen, Liang Fang, Zhe Feng, Guochun Li, Attilio Mucelli, Xu Zhang, Xueping Zhou

**Affiliations:** ^1^The First Clinical Medical College, Nanjing University of Chinese Medicine, Nanjing 210023, China; ^2^School of Health Economics and Management, Nanjing University of Chinese Medicine, Nanjing 210023, China; ^3^College of Medicine and Life Sciences, Nanjing University of Chinese Medicine, Nanjing 210023, China; ^4^Department of Management, School of Economics “Giorgio Fuà”, Università Politecnica delle Marche, No. 8, 60121 Ancona, Italy; ^5^Jiangsu Collaborative Innovation Center of Traditional Chinese Medicine (TCM) Prevention and Treatment of Tumor, Nanjing University of Chinese Medicine, Nanjing 210023, China

## Abstract

*Tripterygium wilfordii* Hook.f. (TWHF) is a traditional Chinese herb long used for rheumatoid arthritis (RA) treatment, in modern times, often in the form of various* Tripterygium wilfordii* Hook.f. preparations (TWPs). This systematic review and meta-analysis focuses on analyzing the clinical efficacy and safety of TWPs in the treatment of RA. Databases were searched to collect the randomized controlled trials (RCTs) on TWPs treating RA published on or before April 10, 2017. Data from 11 studies were included in this meta-analysis. Compared with the control group, TWPs can increase effectiveness, while decreasing erythrocyte sedimentation rate (ESR), rheumatoid factor (RF), C-reactive protein (CRP), and risk of adverse events. TWPs treatment was also more effective than treatment by conventional western medicine (CWM) and Chinese patent medicine or placebo (COP). TWPs significantly decreased the risk of adverse events compared with the CWM group, but not compared with the COP group. Current evidence shows that TWPs are more effective than other western or Chinese medicines we included in this meta-analysis for RA treatment with relatively lower toxicity.

## 1. Introduction 

Rheumatoid arthritis (RA) is a common chronic inflammatory disease that is difficult to treat satisfactorily using western medicine [1]. The descriptive epidemiology of RA is suggestive of a genetic effect. The incidence of RA is higher in Europe than in China, with a prevalence of 0.5–1.0% reported in several European countries [2–10]. As the disease is both chronic and progressive, resultant disabilities and occupational impact pose major clinical challenges [11, 12]. In China, 48% of RA patients experience impairments in their work capacity, often in addition to social capacities [13]. In Finland, Netherlands, UK, Canada, and USA, 20–70% of patients become work-disabled within 5–10 years after symptom onset, with a 50% probability within 4.5–22 years [14, 15]. At present, effective and cost-effective treatment methods are still urgently required.

*Tripterygium wilfordii* Hook.f. (TWHF) is a traditional Chinese herb grown in the east and south of China, Japan, and Korea [16]. It has long been used in traditional Chinese medicine, often for rheumatoid arthritis [17]. TWHF has exhibited multiple pharmacological activities, such as anti-inflammatory, immune modulation, antitumor, and antifertility activities [18]. Specifically regarding its use for RA, numerous preclinical studies have demonstrated immune-suppressive, cartilage protective, and anti-inflammatory effects [19]. Currently, several* Tripterygium wilfordii* Hook.f. preparations (TWPs) derived from TWHF extracts are available, including* Tripterygium wilfordii* tablets and* Tripterygium wilfordii* glycosides tablets. However, the clinical application of TWHF is limited by its narrow therapeutic window and potentially severe toxicity toward several organs including liver and kidney [20]. The most frequent side effects of TWHF are gastrointestinal tract disturbances (especially diarrhea), leukopenia, thrombocytopenia, rash, skin pigmentation, and dysfunctions of the male and female reproductive system [19]. This systematic review and meta-analysis aimed to evaluate the efficacy and safety of TWPs in comparison with other western or Chinese medicines to provide more reliable evidence for the further study and clinical application.

## 2. Methods

### 2.1. Criteria for Considering Studies for This Review

#### 2.1.1. Inclusion and Exclusion Criteria

Studies that met the following criteria were included in the review: (1) published in English or Chinese language; (2) randomized or quasirandomized clinical trial; (3) participating patients having a confirmed diagnosis of RA; (4) TWPs of any kind being used.

Studies that met the following criteria were excluded: (1) randomized crossover trials, case reports, case series, reviews, qualitative studies, or animal experiments; (2) participants being restricted to special populations (e.g., the elderly, juveniles); (3) TWPs interventions being combined with other internal medicines for RA; (4) interventions being in decoction form, not in processed preparations; (5) studies which used TWPs in both the treatment and control groups.

#### 2.1.2. Outcome Measures

The primary outcomes analyzed in this meta-analysis were effectiveness and adverse events. Effectiveness was calculated from the number of patients cured, markedly improved, and improved. Adverse events were calculated from the number of patients who had adverse events and were used to evaluate the safety of TWPs. Secondary outcomes analyzed for this meta-analysis were erythrocyte sedimentation rate (ESR), rheumatoid factor (RF), and C-reactive protein (CRP). All data was taken directly from the original studies.

### 2.2. Literature Search Strategy

Three Chinese language databases and five English language databases were widely searched for all relevant results until April 10, 2017. The Chinese language databases were China National Knowledge Infrastructure (CNKI), VIP Database (VIP), and Wanfang Data. The five English language databases are PubMed, ScienceDirect, Foreign Medical Retrieval System (FMRS), ClinicalTrials.gov, and Cochrane Library. The literature search strategy used the following terms: English (“*Tripterygium wilfordii* Hook f” OR “lei gong teng” OR “thunder god vine” OR “yellow vine”) AND (“rheumatoid arthritis” OR “RA”); and related Chinese (“lei gong teng” OR “huang teng”) AND (“lei feng shi guan jie yan” OR “lei feng shi” OR “guan jie yan” OR “bi zheng” OR “wang bi” OR “jiu bi” OR “li jie” OR “lei feng shi xing guan jie yan”).

### 2.3. Data Collection and Analysis

#### 2.3.1. Data Extraction and Management

Both groups of reviewers (WJ and CN) independently extracted data from the original articles. After checking, any disagreements were settled by discussion between the two groups. All the data were recorded using a data collection form. The form contents were as follows: Source, Eligibility, Methods (study design, total study duration, sequence generation, allocation sequence concealment, and blinding, other concerns about bias), Participants (total number, setting, diagnostic criteria, age, sex, and country), Interventions and controls (total number, specific details), Outcomes (time points, outcome definition, and unit of measurement), Results (number of participants, sample size, missing participants, and summary data), and Miscellaneous information. The collected outcome data was inputted into Review Manager 5.3 (RevMan5.3).

#### 2.3.2. Assessment of Risk of Bias

Assessment of risk of bias was based on random sequence generation (selection bias), allocation concealment (selection bias), blinding of participants and personnel (performance bias), blinding of outcome assessment (detection bias), incomplete outcome data (attrition bias), selective reporting (reporting bias), and other potential sources of bias. Criteria for judging risk of bias were taken from the “risk of bias” assessment tool in The Cochrane Handbook for Systematic Reviews of Interventions 5.1.0. Studies were assessed as “low risk,” “unclear risk,” or “high risk,” with the last category indicating either lack of information or uncertainty over the potential for bias. This judgement was evaluated by two groups (WJ and CN) independently, and disagreements were resolved by a third group (ZXP).

#### 2.3.3. Assessment of Heterogeneity

Heterogeneity was assessed by visually inspecting forest plots and formally estimated by Cochran's *Q* test, in which chi-square distribution is used to make inferences regarding the null hypothesis of homogeneity (*P* < 0.10 was deemed to be representative of statistically significant heterogeneity). We also quantified heterogeneity with *I*^2^ statistic, which measures the degree of inconsistency in the studies by calculating what percentage of the total variation across studies is due to heterogeneity rather than chance. Interpretation is as follows: 0% to 40% might not be important; 30% to 60% may represent moderate heterogeneity; 50% to 90% may represent substantial heterogeneity; 75% to 100% may represent considerable heterogeneity. The importance of the observed value of *I*^2^ depends on the magnitude and direction of effects and the strength of evidence for heterogeneity. A fixed effects model was used when *I*^2^ < 50%; otherwise the random effects model was used.

#### 2.3.4. Data Synthesis

We used RevMan 5.3 for statistical analysis. The extracted data were divided into dichotomous and continuous variables. Data were summarized using risk ratio (RR) with 95% confidence intervals (CI) for dichotomous outcomes; mean difference (MD) with 95% CI was presented for continuous outcomes.

#### 2.3.5. Sensitivity Analysis

For different outcome measures, we investigated possible clinical causes by conducting subgroup and sensitivity analyses. Various subgroup analyses were performed based on types of medication. Sensitivity analyses were performed by removing each study in sequence and recalculating the results, aiming to assess whether one or more of the studies influenced the overall results.

## 3. Results

### 3.1. Results of the Search

**Figure 1:**
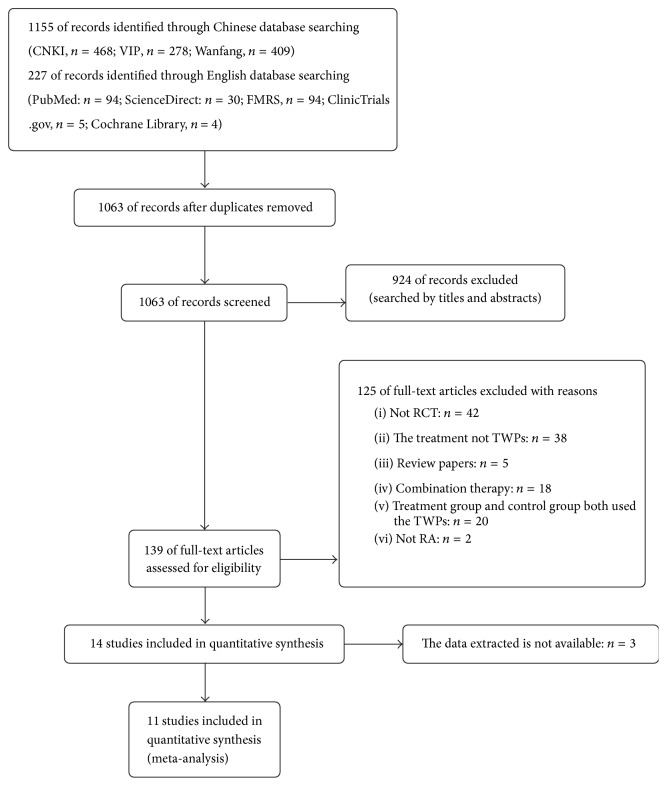
Flowchart of the trial selection process.

The database search obtained 1382 potentially relevant records (1155 records from Chinese databases and 227 records from English databases). 1063 records remained after removal of duplicates. A total of 924 trials were excluded after reading of the titles and abstracts, due to lack of relevance. The full texts of the remaining 139 articles were read and analyzed in detail, with 14 papers finally included for the systematic review. However, 3 of them did not have the available data and were thus also excluded. This screening process is summarized in a flow diagram (Figure 1).

### 3.2. Study Characteristics

The included studies were published between 2009 and 2016. Ten studies were published in Chinese, while one [21] study was in English. All of the randomized controlled trials (RCTs) originated in China and demonstrated no significant difference between control and treatment groups in baseline characteristics. Mean age ranged between 35.8 and 51.3 years, although two studies [22, 23] did not mention age. Mean disease duration ranged from 42 to 120 months, though was unmentioned in three studies [22–24]. Ten studies used the 1987 American Rheumatism Association (ARA 1987) diagnostic criteria, including one [22] combined with the ACR 2009 and the European League Against Rheumatism (2009 ACR/EULAR), while one [21] used the ACR 2010 and the European League Against Rheumatism (2010 ACR/EULAR) criteria. Five [21–23, 25, 26] studies were aimed at active RA, while six studies were not or did not mention it. Eight studies compared TWPs treatment with conventional western medicine, Leflunomide (LEF) [22, 25, 26], Methotrexate (MTX) [21, 23, 24, 27], and Diclofenac Sodium Sustained Release Capsules (DSSRC) [24, 28], one [29] compared it to placebo, and two [30, 31] compared it with other Chinese patent medicines. Eight studies reported participant withdrawal and adverse effects, while three [23, 26, 31] did not. The study characteristics are shown in Table 1.

### 3.3. Risk of Bias

**Figure 2:**
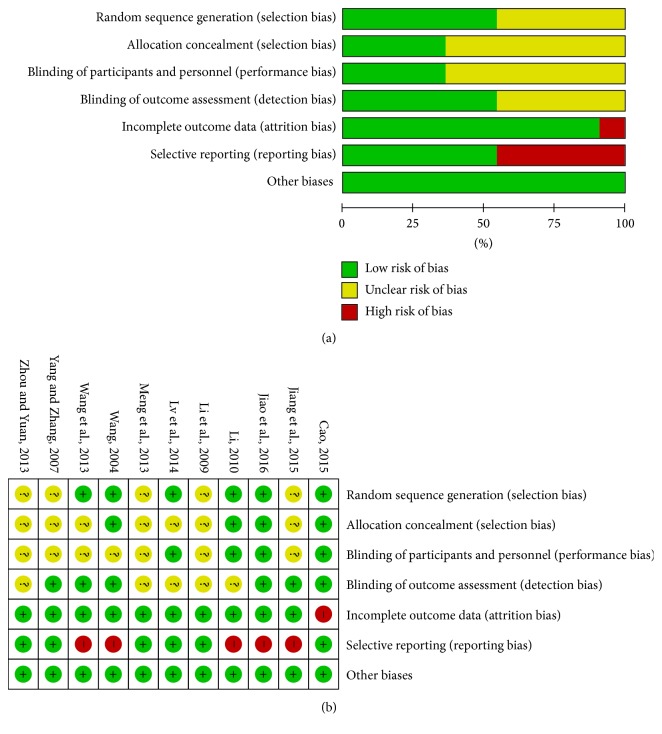
(a) Risk of bias graph: review authors' judgements about each risk of bias item presented as percentages across all included studies. (b) Risk of bias summary: review authors' judgements about each risk of bias item for each included study.

Six [21, 23, 25, 26, 29, 31] of the included articles described the specific method of randomization. Four [23, 25, 29, 31] of them described the allocation concealment method. Four [21, 23, 25, 29] of them stated clearly that participants and personnel were blinded. Six [22, 25–27, 29, 31] of them stated blinding of outcome assessment clearly. Only one [25] had incomplete outcome data. Five [22, 23, 26, 29, 31] studies lacked data for some of our reviewed outcomes. The risk of bias is shown in Figure 2.

### 3.4. Effects of Interventions

#### 3.4.1. Effectiveness

**Figure 3:**
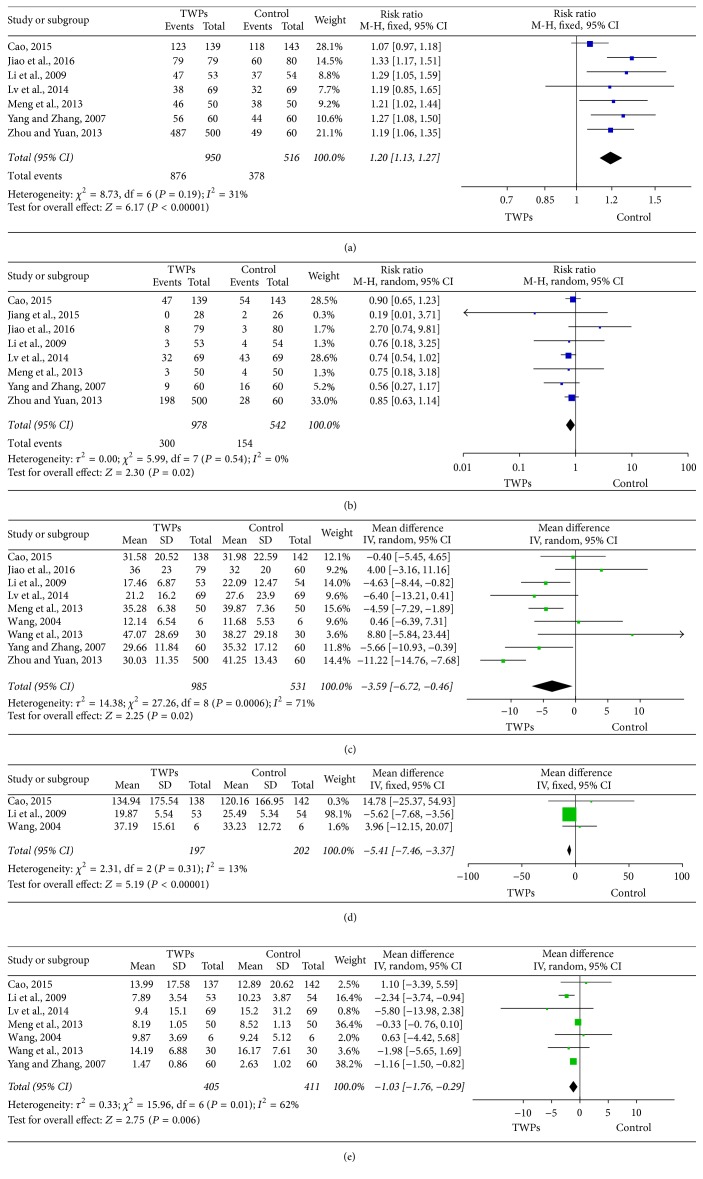
Forest plot of comparison: TWPs group versus control group. (a) Effectiveness. (b) Adverse events. (c) ESR. (d) RF. (e) CRP.

**Figure 4:**
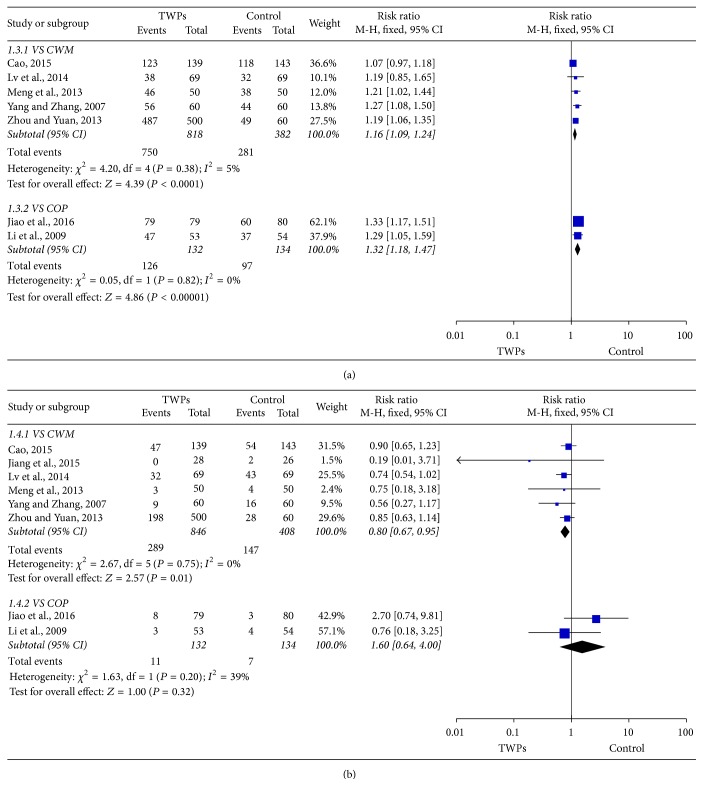
Forest plot of subgroup comparison: TWPs group versus CWM group; TWPs group versus COP group. (a) Effectiveness. (b) Adverse events.

Seven studies evaluated the effectiveness of TWPs with 950 patients in the TWPs group and 516 patients in the control group. Our analysis revealed that TWPs can increase effectiveness compared with the control group (RR: 1.20; 95% CI: 1.13–1.27;* P *< 0.00001). As homogeneity might not be important in the trial results (*χ*^2^ = 8.73;* P *= 0.19; *I*^2^ = 31%), a fixed effects model was applied (Figure 3). Sensitivity analyses were performed to assess the stability of the meta-analysis. The RR ranged from 1.18 to 1.25, indicating a good stability of the meta-analysis (Table 2). Subgroup analyses were divided into a conventional western medicine (CWM) group and a Chinese patent medicine or placebo (COP) group. The result of subgroup analysis revealed that TWPs can increase effectiveness compared with the CWM group (RR: 1.16; 95% CI: 1.09–1.24;* P *< 0.0001), as well as the COP group (RR: 1.32; 95% CI: 1.18–1.47;* P *< 0.00001) (Figure 4).

#### 3.4.2. Adverse Events

Eight studies evaluated the effectiveness of TWPs with 478 patients in the TWPs group and 482 patients in the control group. Our analysis revealed that TWPs can decrease adverse events compared with the control group (RR: 0.82; 95% CI: 0.70–0.97;* P *= 0.02). As the homogeneity might not be important in the trial results (*χ*^2^ = 5.99;* P *= 0.54; *I*^2^ = 0%), a fixed effects model was applied (Figure 3). The RR ranged from 0.80 to 0.86, indicating a good stability of the meta-analysis (Table 2). The result of subgroup analysis revealed that TWPs can decreases adverse events compared with the CWM group (RR: 0.08; 95% CI: 0.67–0.95;* P *= 0.01), but there was no strong evidence in comparing with the COP group (RR: 1.60; 95% CI: 0.64–4.00;* P *= 0.32) (Figure 4).

#### 3.4.3. ESR

Nine studies evaluated ESR of TWPs with 950 patients in the TWPs group and 516 patients in the control group. Our analysis revealed that TWPs can decrease the ESR compared with the control group (MD: −3.59; 95% CI: −6.72–−0.46;* P *= 0.02). As the homogeneity may represent substantial heterogeneity in the trial results (*χ*^2^ = 14.38;* P *= 0.0006; *I*^2^ = 71%), a random effects model was applied (Figure 3). The MD ranged from −3.15 to −4.09, indicating a good stability of the meta-analysis (Table 2).

#### 3.4.4. RF

Three studies evaluated RF of TWPs with 197 patients in the TWPs group and 202 patients in the control group. Our analysis revealed that TWPs can decrease the RF compared with the control group (MD: −5.41; 95% CI: −7.46–−3.37;* P *< 0.00001). As the homogeneity might not be important in the trial results (*χ*^2^ = 2.31;* P *= 0.31; *I*^2^ = 13%), a fixed effects model was applied (Figure 3). The sensitivity analysis showed that MD ranged from −5.41 to 5.46, indicating not a good stability of the meta-analysis (Table 2).

#### 3.4.5. CRP

Seven studies evaluated CRP of TWPs with 405 patients in the TWPs group and 411 patients in the control group. Our analysis revealed that TWPs can decrease the CRP compared with the control group (MD: −1.03; 95% CI: −1.76–−0.29;* P *= 0.006). As the homogeneity may represent substantial heterogeneity in the trial results (*χ*^2^ = 0.33;* P *= 0.01; *I*^2^ = 62%), a random effects model was applied (Figure 3). The sensitivity analysis showed that MD ranged from −0.77 to −1.22, indicating a good stability of the meta-analysis (Table 2).

## 4. Discussion

This meta-analysis included 11 RCTs with 1675 participants, analyzing the effectiveness, adverse events, serology index RF, and acute phase reactants ESR and CRP. The results of the meta-analysis suggest that patients with RA may benefit from TWPs. TWPs can increase effectiveness by 20%, while decreasing the risk of adverse events by 18%. Improvements in ESR, RF and CRP values were dramatic (resp., 359%, 541% and 103% of the improvements seen in control groups). Subgroup analyses were also used for comparing TWPs versus CWM and TWPs versus COP. These showed that TWPs can increase effectiveness by 16% compared with the CWM group and 32% compared with COP. TWPs can decrease the risk of adverse events by 20% compared with the CWM group, but no strong evidence appeared for a decrease in risk compared with COP.

Previous meta-analyses on TWHF focused on treatment using the whole herb, or its extract, whereas the present analyzed only results from preparations (TWPs). Our results echoed other systematic reviews and meta-analyses that focused on TWHF herbal treatment. TWHF extracts have been found to reduce RA signs and symptoms such as ESR, RF, CRP, grip strength, and 15m walking time, compared to certain drugs or placebos [32–35]. However, our adverse events comparison differed from Jiang et al. [34], in which the incidence of adverse reactions in* Tripterygium wilfordii* extract (TWE) treatment was higher than with Disease-modifying Antirheumatic Drugs (DMARDs). In contrast, the DMARDs, Leflunomide (LEF) and Methotrexate (MTX), were included in our meta-analysis control group and showed more frequent adverse events than the TWPs group. In our review, the most frequent adverse effects of TWPs were gastrointestinal discomfort, skin and mucous events, and menstruation disorders (Table 3). These frequencies likely differed from Liu et al. [32] as we included a study of a topical agent. Therefore, the skin and mucous events may have not been directly just caused by TWPs, but by other materials used in the topical preparations. Regardless, incidences of skin and mucous events were still lower than the control group in this review. Of course, it may be due to the different trails the two reviews included or to the low quality of the trails the present review included which might have report bias.

In conventional medicine, RA is often managed with Nonsteroidal Anti-inflammatory Drugs (NSAIDs), DMARDs, and biological agents. NSAIDs are unable to modify the long-term course of disease and have toxic gastrointestinal and cardiac effects [36, 37]. DMARD use carries risks such as hepatotoxicity, blood dyscrasias, and interstitial lung disease [38, 39]. Biological agents are under a higher a priori risk of infection, and the cost-effectiveness of very early intervention remains uncertain [40, 41].

Management of rheumatoid arthritis should be effective and affordable [36]. According to Finckh et al.'s research, the cost-effectiveness ratio of the early DMARD strategy is $4849 per QALY (quality-adjusted life-year) [41]. As herbal preparations are often cheaper than pharmaceutical drugs, it is likely that TWPs would be a more economical treatment than conventional medicine, and at least effective. If so, introducing TWPs into conventional RA treatment can potentially provide a significant cost-saving measure. A precise estimate of the financial benefit would require the further research.

A few limitations in this meta-analysis should be considered. First, the baseline physical activity of the two groups may be different and may be a potential confounding factor. Second, the results for ESR and CRP have significant heterogeneity, perhaps due to widely varying clinical standards, intervention methods, doses, and duration of treatment. Third, the included studies were in general of poor methodological quality, at times lacking information about random sequence generation, allocation concealment, or blinding of participants, personnel, and outcome assessors. Fourth, the longest follow-up period was 24 weeks, which does not allow analysis of long-term toxicity and more serious adverse events, for example, reversible amenorrhea in women and infertility in men.

Recommendations for future research include higher quality clinical studies with a longer follow-up period. A further meta-analysis comparing TWPs combined with DMARDs to TWPs alone would be a further step to build on present results. The standardization of clinical trial procedures would assist greatly in performing comparisons and meta-analyses. Further research into both potential toxicity of and detoxification from TWPs is needed. Last but not least, an analysis of TWPs' cost-effectiveness will help inform policy-makers, physicians, and patients.

## 5. Conclusion

From this meta-analysis, it is evident that TWPs may be efficacious in treating RA with relatively lower toxicity. It is our hope that further research can determine which TWP is the most effective, safe, and cost-effective option for RA patients.

## Figures and Tables

**Table 1:** Trials characteristics.

Study ID	Group	Age (year)	Disease duration (months)	Outcomes	Diagnostic criteria	Active or not	Duration (week)	Intervention group			Control group			Withdrawal/adverse effects
								Patients *N* (M/F)	Meditation	Using method	Patients *N* (M/F)	Meditation	Using method	
Cao, 2015	TWPs CWM	47.81 (10.37) 48.94 (10.51)	59.39 (67.11) 61.80 (65.62)	ACR 20/50/70, DAS28, ESR, CRP, RF, CCP, GP, VAS, DMS, TJC, SJCHAQ, QLT, SDS, SAS, X ray	ARA 1987	Y	12	139 (17/122)	XFC LEF PLB	3 tid po 10 mg qd po	143 (27/116)	LEF XFC PLB	10 mg qd 3 tid	Y

Jiang et al., 2015	TWPs CWM	18–75	-	VAS, DAS28, AOT, SOT	ARA 1987 ACR/EULAR 2009	Y	12	28	KXC	2 tid po	28	LEF	20 mg qd	Y

Jiao et al., 2016	TWPs PLB	52 (9) 52 (10)	120 (96) 102 (77)	VAS, DAS28, ESR, hs-CRP	ARA 1987	-	8	87 (6/81)	FFLTA	20 g qd ext	87 (14/73)	PLB	20 g qd ext	Y

Li, 2010	TWPs CWM	-	-	TJC, SJC, DMS, QLT, ESR, CRP	ARA 1987	Y	12	33	LJS MTX PLB	66 g tid po 15 mg qw po	33	MTX LJS PLB	15 mg qw po 66 g tid po	N

Li et al., 2009	TWPs CPM	44.6 (13.1) 43.6 (14.5)	68.88 (87.12) 66.48 (87.48)	HAQ, DMS, VAS, TJC, SJC, CRP, ESR, RF	ARA 1987	-	8	53 (15/38)	STTBW ZQFTN PLB	4 g bid po 40 mg tid po	54 (12/42)	ZQFTN STTBW PLB	40 mg tid po 4 g bid po	Y

Lv et al., 2014	TWPs CWM	51.3 (8.3) 51.0 (10.3)	61.9 (81.9) 58.8 (88.7)	PAP, PGADA, PGADA, TJC, SJC, ESR, CRP, HAQ, SF-36, DAS28	ACR/EULAR 2010	Y	24	69 (13/56)	TWHF pills MTX	20 mg tid po	69 (10/59)	MTX	7.5–12.5 mg qw po	Y

Meng et al., 2013	TWPs CWM	38.25 (4.43) 39.33 (3.58)	-	TJC, SJC, SC, VAS, DMS, MGS, 15 mWT	ARA 1987	N	12	50 (11/39)	FFLGTYJ	10 ml tid po with honey	50 (9/41)	MTX DSSRC	10 mg qw po 50 mg bid po	Y

Wang, 2004	TWPs CPM	42.64 (14.52) 41.87 (11.96)	94.44 (28.08) 91.56 (26.28)	DMS, MGS, VAS, SJC, TJC, ESR, CRP, RF, IgG, IgM, IgA	ARA 1987	N	12	6 (3/3)	TWHF pills	10 mg tid po	6 (2/4)	YSJBW	8 g tid po	N

Wang et al., 2013	TWPs CWM	47.43 (8.75) 43.67 (10.10)	67.10 (54.02) 67.93 (56.95)	TJC, SJC, DMS, ESR, CRP, RF, IgG	ARA 1987	Y	4	30 (3/27)	XFC	3 tid po	30 (2/28)	LEF	10 mg qd po	N

Yang and Zhang, 2007	TWPs CWM	37.6 35.8	42 45.6	DMS, TJC, SJC, MGS, 20 mWT, ESR, CRP, RF, IgG, IgM, IgA	ARA 1987	N	4	60 (18/42)	TWHF pills	20 mg tid po	60 (16/44)	MTX	15 mg qw po	Y

Zhou and Yuan, 2013	TWPs CWM	37.28 37.25	78.24 78	DMS, TJC, SJC, MGS, 20 mWT, ESR, CRP, RF	ARA 1987	N	12	500 (123/377)	LDJ	20 ml tid po	60 (15/45)	DSSRC	50 mg bid po	Y

*Note*. TWPs: *Tripterygiumwilfordii* Hook.f. preparations; CWM: conventional western medicine; CPM: Chinese patent medicine; PLB: placebo; N: no; Y: yes; -: not mentioned; XFC: Xin Feng capsules; LEF: Leflunomide; KXC: Kun Xian capsules; FFLTA: Fu Fang Lei Gong Teng topical agent; LJS: Lei Gong Teng Jia Su; MTX: Methotrexate; STTBW: San Teng Tong Bi Wan; ZQFTN: Zheng Qing Feng Tong Ning; TWHF pills: Lei Gong Teng Duo Dai pills; LDJ: Lei Gong Teng Dang Gui wine; YSJBW: Yi Shen Juan Bi Wan; DSSRC: Diclofenac Sodium Sustained Release Capsules; ACR20/50/70: American College of Rheumatology Criteria 20/50/70; DAS28: Disease Activity Score 28; ESR: erythrocyte sedimentation rate; CRP: C-reactive protein; RF: rheumatoid factor; CCP: cyclic citrullinated peptide (CCP) antibody; hs-CRP: high-sensitivity CRP; VAS: Visual Analogue Scale; DMS: duration of morning stiffness; TJC: tender joint count; SJC: swollen joint count; MGS: mean grip strength; 15 mWT: 15 m walking time; 20 mWT: 20 m walking time; AOT: analgesic onset time; SOT: swelling onset time; SF-36: short form 36 health questionnaire; HAQ: Health Assessment Questionnaire.

**Table 2:** The results of the included studies through sensitivity analysis.

Excluded study	TWPs group (number)	Control group (number)	RR/MD (95% CI)	*P* value	Heterogeneity test	Effect model
*Effectiveness*						
Before excluding	950	516	1.20 [1.13, 1.27]	*P* < 0.00001	*P* = 0.19, *I*^2^ = 31%	Fixed
Cao, 2015	811	373	1.25 [1.16, 1.34]	*P* < 0.00001	*P* = 0.86, *I*^2^ = 0%	Fixed
Jiao et al., 2016	871	436	1.18 [1.10, 1.25]	*P* < 0.00001	*P* = 0.37, *I*^2^ = 7%	Fixed
Li et al., 2009	897	462	1.19 [1.12, 1.26]	*P *< 0.00001	*P* = 0.15, *I*^2^ = 38%	Fixed
Lv et al., 2014	881	447	1.20 [1.13, 1.27]	*P *< 0.00001	*P* = 0.12, *I*^2^ = 43%	Fixed
Meng et al., 2013	900	466	1.20 [1.13, 1.27]	*P *< 0.00001	*P* = 0.12, *I*^2^ = 43%	Fixed
Yang and Zhang, 2007	890	456	1.19 [1.12, 1.26]	*P* < 0.00001	*P* = 0.15, *I*^2^ = 38%	Fixed
Zhou and Yuan, 2013	450	456	1.20 [1.12, 1.28]	*P *< 0.00001	*P* = 0.12, *I*^2^ = 43%	Fixed
*Toxicity*						
Before excluding	978	542	0.83 [0.70, 0.98]	*P* = 0.03	*P* = 0.54, *I*^2^ = 0%	Fixed
Cao, 2015	839	399	0.80 [0.65, 0.98]	*P* = 0.03	*P* = 0.47, *I*^2^ = 0%	Fixed
Jiang et al., 2015	950	516	0.84 [0.71, 0.99]	*P* = 0.04	*P* = 0.54, *I*^2^ = 0%	Fixed
Jiao et al., 2016	899	462	0.80 [0.67, 0.95]	*P *= 0.009	*P* = 0.85, *I*^2^ = 0%	Fixed
Li et al., 2009	925	488	0.83 [0.70, 0.99]	*P *= 0.03	*P* = 0.42, *I*^2^ = 0%	Fixed
Lv et al., 2014	909	473	0.86 [0.70, 1.05]	*P* = 0.13	*P* = 0.49, *I*^2^ = 0%	Fixed
Meng et al., 2013	928	492	0.83 [0.70, 0.99]	*P *= 0.03	*P* = 0.43, *I*^2^ = 0%	Fixed
Yang and Zhang, 2007	918	482	0.86 [0.72, 1.02]	*P* = 0.08	*P* = 0.55, *I*^2^ = 0%	Fixed
Zhou and Yuan, 2013	478	482	0.82 [0.67, 1.01]	*P *= 0.06	*P* = 0.43, *I*^2^ = 0%	Fixed
*ESR*						
Before excluding	985	531	−3.59 [−6.72, −0.46]	*P* = 0.02	*P* = 0.0006, *I*^2^ = 71%	Random
Cao, 2015	847	389	−4.02 [−7.37, −0.68]	*P* = 0.02	*P* = 0.001, *I*^2^ = 71%	Random
Jiao et al., 2016	906	471	−4.46 [−7.48, −1.44]	*P* = 0.004	*P* = 0.004, *I*^2^ = 67%	Random
Li et al., 2009	932	477	−3.25 [−6.97, 0.46]	*P* = 0.09	*P* = 0.0003, *I*^2^ = 74%	Random
Lv et al., 2014	916	462	−3.22 [−6.64, 0.21]	*P* = 0.07	*P* = 0.0003, *I*^2^ = 74%	Random
Meng et al., 2013	935	481	−3.15 [−7.10, 0.79]	*P* = 0.12	*P* = 0.0003, *I*^2^ = 74%	Random
Wang, 2004	979	525	−4.02 [−7.30, −0.73]	*P* = 0.02	*P* = 0.0008, *I*^2^ = 72%	Random
Wang et al., 2013	955	501	−4.09 [−7.15, −1.03]	*P* = 0.009	*P* = 0.001, *I*^2^ = 71%	Random
Yang and Zhang, 2007	925	471	−3.21 [−6.74, 0.32]	*P* = 0.08	*P* = 0.0003, *I*^2^ = 74%	Random
Zhou and Yuan, 2013	485	471	−3.38 [−5.08, −1.69]	*P* < 0.0001	*P* = 0.10, *I*^2^ = 41%	Fixed
*RF*						
Before excluding	197	202	−5.41 [−7.46, −3.37]	*P* < 0.00001	*P* = 0.31, *I*^2^ = 13%	Fixed
Cao, 2015	59	60	−5.47 [−7.51, −3.42]	*P* < 0.00001	*P* = 0.25, *I*^2^ = 25%	Fixed
Li et al., 2009	144	148	5.46 [−9.49, 20.41]	*P* = 0.47	*P *= 0.62, *I*^2^ = 0%	Fixed
Wang, 2004	191	196	−5.57 [−7.63, −3.51]	*P* < 0.00001	*P *= 0.32, *I*^2^ = 0%	Fixed
*CRP*						
Before excluding	405	411	−1.03 [−1.76, −0.29]	*P* = 0.006	*P* = 0.01, *I*^2^ = 62%	Random
Cao, 2015	268	269	−1.08 [−1.83, −0.33]	*P* = 0.005	*P* = 0.010, *I*^2^ = 67%	Random
Li et al., 2009	352	357	−0.77 [−1.48, −0.06]	*P* = 0.03	*P* = 0.04, *I*^2^ = 57%	Random
Lv et al., 2014	336	342	-0.99 [-1.71, -0.26]	*P* = 0.008	*P* = 0.01, *I*^2^ = 66%	Random
Meng et al., 2013	355	361	-1.22 [-1.54, -0.89]	*P* < 0.00001	*P* = 0.36, *I*^2^ = 9%	Fixed
Wang, 2004	399	405	-1.07 [-1.83, -0.31]	*P* = 0.006	*P* = 0.008, *I*^2^ = 68%	Random
Wang et al., 2013	375	381	-0.99 [-1.76, -0.23]	*P* = 0.01	*P* = 0.008, *I*^2^ = 68%	Random
Yang and Zhang, 2007	345	351	-1.08 [-2.44, 0.27]	*P* = 0.12	*P* = 0.07, *I*^2^ = 51%	Random

**Table 3:** Adverse events reported.

Adverse events (*n*)	TWPs (*n* = 789)	All of control group drugs (*n* = 349)	LEF (*n* = 26)	MTX (*n* = 129)	PLB (*n* = 80)	ZQFTN (*n* = 54)	DSSRC (*n* = 60)
All, *n* (%)	264 (33.46)	137 (39.26)	2 (7.69)	100 (77.52)	3 (3.75)	4 (7.41)	28 (46.67)
Gastrointestinal, *n* (%)	208 (26.36)	63 (18.05)	0	38 (29.46)	0	4 (7.41)	21 (35)
Blood and lymphatic system disorders, *n* (%)	0	5 (1.43)	1 (3.85)	4 (3.10)	0	0	0
Infection, *n* (%)	3 (0.38)	10 (2.87)	0	10 (7.75)	0	0	0
Hepatic function abnormality, *n* (%)	10 (1.27)	16 (4.58)	1 (3.85)	12 (9.30)	1 (1.25)	0	2 (3.33)
Renal function abnormality, *n* (%)	0	3 (0.86)	0	3 (2.33)	0	0	0
Skin and mucous event, *n* (%)	17 (2.15)	20 (5.73)	0	14 (10.85)	2 (2.5)	0	4 (6.67)
Irregular menstruation, *n* (%)^*∗*^	13 (2.07)	3 (1.08)	0	3 (2.92)	0	0	0
Headache, *n* (%)	1 (0.13)	4 (1.15)	0	3 (2.33)	0	0	1 (1.67)
Angular cheilitis, *n* (%)	7 (0.89)		0	0	0	0	0
Other adverse events, *n* (%)^*∗∗*^	5 (0.63)	13 (3.72)	0	13 (10.08)	0	0	0

*Note*. ^*∗*^The percentages of irregular menstruation were based on the calculated total number of female patients in each group. The calculation methods were based on female patients account for 79.65% of the total number. ^*∗∗*^Other adverse events including fatigue, weight loss, anemia, palpitations, haematuria, peripheral oedema, hair loss, and unbearable smell.

## Abbreviations

TWHF: * Tripterygium wilfordii* Hook.f.
RA: Rheumatoid arthritis
TWPs: * Tripterygium wilfordii* Hook.f. preparations
CWM: Conventional western medicine
COP: Chinese patent medicine or placebo
ESR: Erythrocyte sedimentation rate
RF: Rheumatoid factor
CRP: C-reactive protein
RR: Risk ratio
MD: Mean difference
CI: Confidence intervals.
